# Pott’s Puffy Tumor: A Case Report of a Rare Complication of Frontal Sinusitis

**DOI:** 10.7759/cureus.110218

**Published:** 2026-06-03

**Authors:** Muniba Manzoor, Muhammad Naveed, Rashid Mahmood, Muzammel Manzoor

**Affiliations:** 1 Radiology, Combined Military Hospital, Rawalpindi, PAK; 2 Radiology, Children's Health Ireland at Crumlin, Crumlin, IRL; 3 Radiology, Bahria International Hospital, Islamabad, PAK

**Keywords:** frontal sinusitis, osteomyelitis, pott’s puffy tumor, ppt, radiology

## Abstract

Among complications of frontal sinusitis, Pott’s puffy tumor (PPT) is one of the rare complications, which is characterized by the occurrence of subperiosteal abscess and osteomyelitis of the frontal bone. Primarily, infection or trauma is the causative agent and is often seen in late childhood and adolescence. If left untreated for a longer period of time, further intracranial complications such as subdural empyema, epidural abscess, cerebral abscess, and meningitis may occur. We report a case of a 13-year-old male who presented with a history of a fall 15 days ago, and the clinical query was to rule out a fracture and subdural hematoma. The primary diagnosis was made based on clinical and radiological findings, highlighting the significance of maintaining a high index of suspicion of this rare condition and the value of CT imaging in the timely diagnosis and management.

## Introduction

Pott’s puffy tumor (PPT) is a severe and rare condition characterized by frontal bone osteomyelitis and a subperiosteal abscess, with potential intracranial spread [1]. It is usually secondary to untreated frontal sinusitis or trauma, and its intracranial complications develop due to the anatomy of the sinuses and their venous drainage [2]. The condition was first described by Percivall Pott in 1768 and was initially associated with head trauma [3]. The pathophysiology involves the spread of infection from the frontal sinus mucosa to the diploic veins of the frontal bone, resulting in osteomyelitis and the subsequent formation of a subperiosteal abscess [4]. If PPT is left untreated, intracranial complications such as subdural empyema, epidural abscess, meningitis, and cerebral abscess may occur [5]. Despite the widespread use of antibiotics, PPT remains clinically relevant because of its potential for rapid progression and serious intracranial complications. Clinically, patients with PPT typically present with forehead swelling, fever, headache, signs of systemic infection, and nasal discharge, although the presentation may vary depending on the extent of disease progression [6].

Diagnostic imaging plays a very important role in the process of diagnosis by aiding in the confirmation of PPT and ruling out complications that may necessitate further treatment [7]. Among imaging modalities, magnetic resonance imaging (MRI) and computed tomography (CT) play a crucial role in the diagnosis, as CT delineates air-bone and soft tissue interfaces as well as bony anatomy, whereas MRI is considered the gold standard for the detection of intracranial or orbital complications and provides superior soft-tissue resolution [8,9]. In addition, management generally requires a combination of prolonged antibiotic therapy and surgical intervention, including debridement of necrotic bone and drainage of the abscess, to prevent further morbidity and mortality [10,11].

Given its rarity and the potential for severe complications, timely diagnosis and management of PPT are crucial for favorable clinical and patient-associated outcomes. The present case study aimed to highlight a rare presentation of this condition.

## Case presentation

A 13-year-old male presented with a history of a fall 15 days ago, headache, vomiting, swelling of the left eye and left forehead for 2 days, and local tenderness. Clinical examination showed localized erythematous swelling over the forehead, tenderness over the frontal sinus, and intact extraocular movements without pain. The clinical query was to rule out a subdural hematoma. Neurological examination was normal, with intact cranial nerves; no nystagmus or ophthalmoplegia was present. Most laboratory tests were within normal limits, except for white blood cell count, which was elevated (11,500 cells/mcL), as described in Table 1.

**Table 1:** Laboratory parameters.

Labs	Values	Normal range	Status
Hemoglobin	14 g/dL	13.5-17.5 g/dL	Normal
Neutrophil	52%	40%-70%	Normal
White blood cells	11,500 cells/mcL	4,000-11,000 cells/mcL	Elevated
Platelets count	210,000/mcL	150,000-450,000/mcL	Normal
C-reactive protein	3.3 mg/L	<5 mg/L	Normal

CT imaging was performed without intravenous contrast using a routine protocol. Axial CT brain images showed opacification of both frontal sinuses with a soft tissue collection (Figures 1A, 1B).

**Figure 1:**
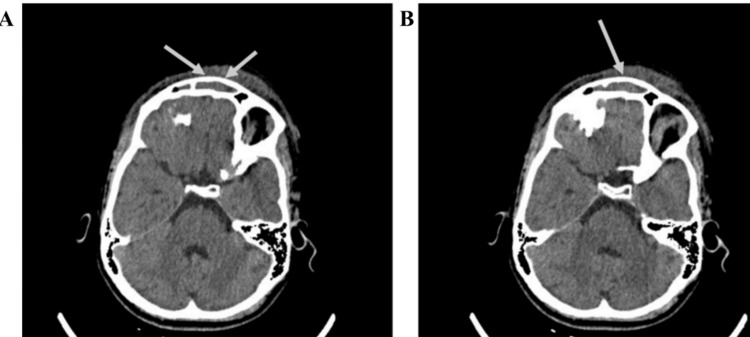
Axial non-contrast CT images of the brain. (A) and (B) Opacification of both frontal sinuses with a soft tissue collection.

Axial CT images indicated focal erosion of the left frontal bone with irregular destruction and marked thinning of both the inner and outer tables. The cortical margins appeared disrupted, with areas of reduced bone density and loss of normal trabecular architecture, findings highly suggestive of underlying osteomyelitis. Such changes can reflect the spread of infection from the frontal sinus to the adjacent calvarium and are characteristic of the osseous involvement seen in PPT (Figure 2).

**Figure 2:**
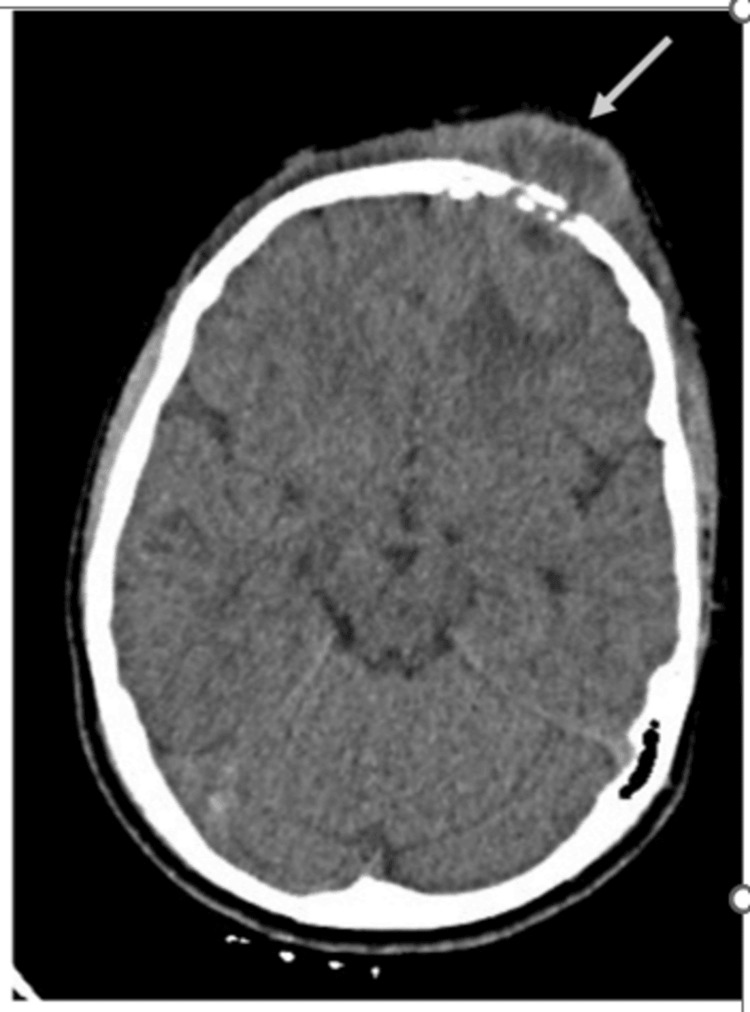
Axial CT bone window shows bone erosion of the left frontal bone and thinning of the inner and outer table (arrow), suggesting osteomyelitis.

Furthermore, axial CT imaging demonstrated a hypodense collection within the soft tissues of the forehead as well as an epidural abscess in the left frontal region (Figure 3A). Sagittal CT of the brain corroborated these findings, redemonstrating both epidural and subperiosteal abscess formation over the left frontal region. In addition, a hypodense area within the adjacent frontal lobe parenchyma was identified, raising concern for intracranial extension and early intracerebral involvement in the context of an evolving infectious process (Figure 3B).

**Figure 3:**
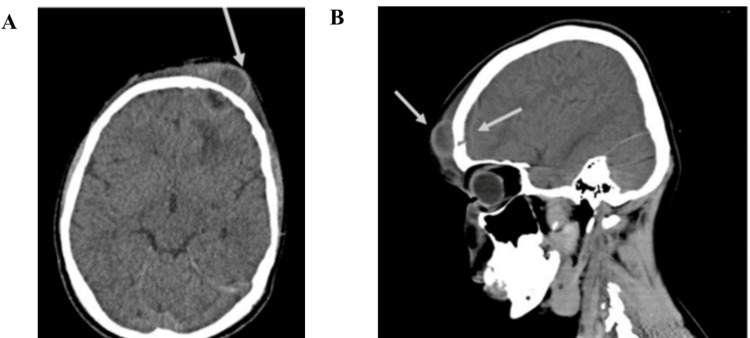
(A) Axial CT brain image shows an epidural abscess in the left frontal region; (B) sagittal CT image of the brain shows a frontal subperiosteal abscess with intracranial extension.

Based on clinical and radiological findings, the patient was diagnosed with frontal sinusitis complicated by PPT. Meanwhile, routine biochemistry was unremarkable.

Following radiological confirmation of frontal sinusitis complicated by PPT, the patient was managed with prolonged broad-spectrum intravenous antibiotic therapy, initially administered moxifloxacin 400 mg, followed by linezolid 400 mg for up to 10 weeks. This therapeutic approach led to resolution of the abscess, with subsequent clinical improvement, and the patient is now completely recovered without any signs of recurrence. The sequence of events is shown in Figure 4.

**Figure 4:**
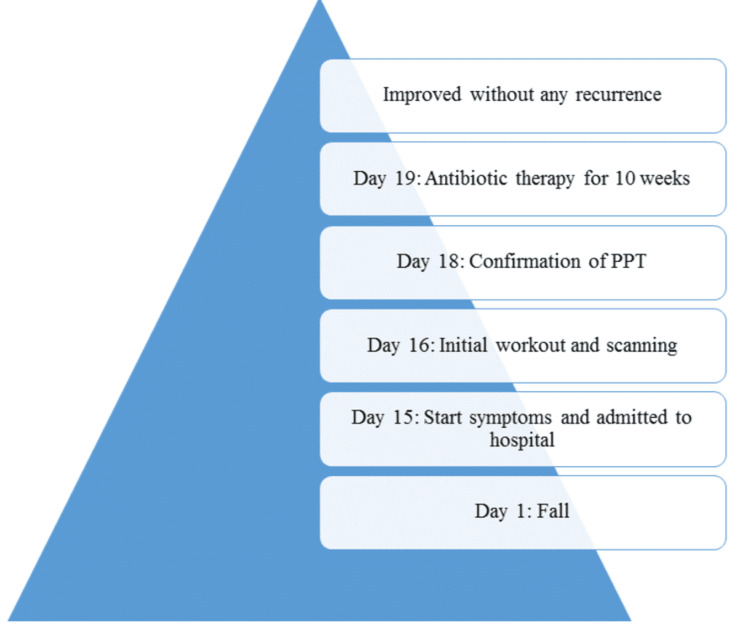
Sequence of events from Day 1 to patient recovery. Image created by the authors using Microsoft PowerPoint. PPT, Pott’s puffy tumor

## Discussion

The present case of a 13-year-old male highlights the importance of maintaining a high index of suspicion for PPT in adolescents presenting with forehead swelling and symptoms of sinusitis. Early radiological evaluation with CT imaging enables timely diagnosis and facilitates appropriate management, including antibiotic therapy and surgical intervention, which leads to favorable clinical outcomes. Meanwhile, PPT develops due to the spread of infection from the frontal sinus to the frontal bone via diploic veins, causing osteomyelitis and subperiosteal abscess formation [5]. The most common causes include frontal sinusitis, trauma, or intranasal drug use such as cocaine abuse, although these are rare [12]. In the pediatric population, the primary cause of PPT is sinusitis, which is associated with increased diploic venous blood flow and incomplete development of the frontal sinuses, leading to an increased risk of spread of infection to the surrounding frontal bone [7]. The most common symptoms include forehead swelling, fever, headache, nasal discharge, rhinorrhea, and periorbital erythema or edema [13,14]. Other symptoms may include vomiting, nausea, cutaneous fistulas, as well as visual and neurological symptoms, depending on the presence or absence of ocular and intracerebral complications [15]. In addition, laboratory examinations are typically useful and sometimes show elevated leukocyte counts; however, in our case, laboratory tests were normal. Therefore, for early diagnosis, the selection of an imaging modality considers various factors, including cost-effectiveness, availability, and technical advantages such as improved contrast resolution and the lack of ionizing radiation [16]. CT can reveal sinusitis, subperiosteal collections, bone erosion, and intracranial extension, with osteomyelitis indicated by low-attenuation areas of lytic bone destruction [17]. Due to these benefits, we performed CT to diagnose PPT, as CT is the first-line imaging modality because it shows bone erosion, sinus opacification, and subperiosteal abscess formation. Meanwhile, MRI is used when intracranial complications are suspected, and it also enables characterization of intra- and extracranial collections using diffusion-weighted sequences [18]. After diagnosis, management of PPT requires a multidisciplinary approach that usually combines intravenous antibiotic therapy and surgical intervention [19]. Meanwhile, antibiotic choice should be dependent on national, regional, or local guidelines and resistance patterns, but should be broad-spectrum, covering anaerobes and gram-positive organisms, and should also be able to penetrate the blood-brain barrier in cases of intracranial involvement [20]. Antibiotic therapy should be individualized based on the degree of intracranial involvement and osteomyelitis [21]. In our case, we used moxifloxacin 400 mg followed by linezolid 400 mg, whereas other physicians have used cefepime and metronidazole along with vancomycin [1]. This indicates that antibiotic therapy should be individualized. Additionally, the surgical approach may include drainage and incision of the subperiosteal abscess, trephination, endoscopic sinus surgery, or a combination of these approaches [20]. Early intervention is crucial to prevent disease progression and reduce morbidity. Despite advances in imaging and treatment, if left untreated, serious complications may occur, including epidural abscess, subdural empyema, brain abscess, or cavernous sinus thrombosis [22]. Therefore, early recognition, prompt imaging, and timely management are essential for the most favorable clinical and patient outcomes.

## Conclusions

Although rare, PPT should be considered in patients with serious complications of frontal sinusitis, which is characterized by a subperiosteal abscess and frontal bone osteomyelitis. This case underscores the importance of early recognition, radiological evaluation, and a multidisciplinary management approach to prevent serious complications, including intracranial abscess formation. For imaging, CT plays a crucial role in diagnosis by clearly demonstrating frontal sinus disease, bony erosion, and associated soft-tissue swelling. Prompt management with broad-spectrum antibiotics and surgical drainage leads to favorable outcomes. Overall, our case highlights the importance of maintaining a high index of suspicion and the value of CT imaging in the timely diagnosis and management of this uncommon condition.
